# The Differences and Changes of Semi-Quantitative and Quantitative CT Features of Coronavirus Disease 2019 Pneumonia in Patients With or Without Smoking History

**DOI:** 10.3389/fmed.2021.663514

**Published:** 2021-09-08

**Authors:** Xingzhi Xie, Zheng Zhong, Wei Zhao, Shangjie Wu, Jun Liu

**Affiliations:** ^1^Department of Radiology, Second Xiangya Hospital, Central South University, Changsha, China; ^2^Department of Radiology, First Hospital of Changsha, Changsha, China; ^3^Changsha Public Health Treatment Center, Changsha, China; ^4^Department of Respiratory Medicine, Second Xiangya Hospital, Central South University, Changsha, China; ^5^Department of Radiology Quality Control Center, Changsha, China

**Keywords:** AI, COVID-19, cigarette smoke, CT images, quantitative CT technique, interstitial lung changes

## Abstract

**Objective:** To assess CT features of COVID-19 patients with different smoking status using quantitative and semi-quantitative technologies and to investigate changes of CT features in different disease states between the two groups.

**Methods:** 30 COVID-19 patients with current smoking status (29 men, 1 woman) admitted in our database were enrolled as smoking group and 56 COVID-19 patients without smoking history (24 men, 32 women) admitted during the same period were enrolled as a control group. Twenty-seven smoking cases and 55 control cases reached recovery standard and were discharged. Initial and follow-up CT during hospitalization and follow-up CT after discharge were acquired. Thirty quantitative features, including the ratio of infection volume and visual-assessed interstitial changes score including total score, score of ground glass opacity, consolidation, septal thickening, reticulation and honeycombing sign, were analyzed.

**Results:** Initial CT images of the smoking group showed higher scores of septal thickening [4.5 (0–5) vs. 0 (0–4), *p* = 0.001] and reticulation [0 (0–5.25) vs 0 (0–0), *p* = 0.001] as well as higher total score [7 (5–12.25) vs. 6 (5–7), *p* = 0.008] with statistical significance than in the control group. The score of reticulation was higher in the smoking group than in the control group when discharged [0.89 (0–0) vs. 0.09 (0–0), *p* = 0.02]. The score of septal thickening tended to be higher in the smoking group than the control group [4 (0–4) vs. 0 (0–4), *p* = 0.007] after being discharged. Quantitative CT features including infection ratio of whole lung and left lung as well as infection ratio within HU (−750, −300) and within HU (−300, 49) were higher in the control group of initial CT with statistical differences. The infection ratio of whole lung and left lung, infection ratio within HU (−750), and within HU (−750, −300) were higher in the control group with statistical differences when discharged. This trend turned adverse after discharge and the values of quantitative features were generally higher in the smoking group than in the control group without statistical differences.

**Conclusions:** Patients with a history of smoking presented more severe interstitial manifestations and more residual lesion after being discharged. More support should be given for COVID-19 patients with a smoking history during hospitalization and after discharge.

## Introduction

Coronavirus Disease 2019 (COVID-19) has spread across the world and the number of confirmed cases is continually rising (1, 2). As of November 24, 2020, 58,712,326 confirmed cases and 1,388,528 death cases from COVID-19 involving 219 countries, areas, or territories had been reported (3). Through thorough research, the epidemiology, clinical symptoms, pathological characteristics, and biological features have been well-established. The development of a vaccine targeting severe acute respiratory syndrome coronavirus 2 (SARS-CoV-2) has also made substantial progress. However, how to prevent COVID-19 patients from lethal medical events, e.g., acute respiratory distress syndrome (ARDS), and to provide medical support for recovered COVID-19 patients after being discharged remain tricky.

Therefore, investigating the risk factors for predicting the outcome of patients with COVID-19 during hospitalization and after discharge is clinically urgent. Previous studies stated that patients of an elderly age and with other disease conditions had worse outcomes (4, 5). A previous study conducted by Hu et.al demonstrated COVID-19 patients with pre-existing chronic obstructive pulmonary disease (COPD) had a higher risk of all-cause mortality (6). Smoking is also reported to be high risk factor for COVID-19 patients (7). Smoking can cause lung injuries, leading to emphysema and fibrosis (8, 9), and is related to higher expression of angiotensin converting enzyme 2 (ACE2), which is the receptor for SARS-CoV-2. So it might also be an independent factor for COVID-19 infection and might worsen the disease prognosis (10). A meta-analysis conducted by Zhao and colleagues discovered that active smoking increases the risk of developing severe COVID-19 by around 2-fold (11). Hence, carefully evaluating the high-risk patients with smoking may facilitate a better treatment scenario.

Computed tomography (CT) has proven to be an important tool in diagnosing and evaluating the response of COVID-19 in clinical practice (12–14). Our previous research (15–17) also discovered that chest CT can be used as a potential tool to diagnose and evaluate the severity of COVID-19. The CT imaging features of COVID-19 patients had been well-described, e.g., bilateral and peripheral distributed ground-glass opacities. Studies also discovered that CT can evaluate the severity and extent of fibrosing interstitial pneumonia (18, 19). However, whether COVID-19 patients with or without smoking history have specific radiographic characteristics is not clear.

With the state-of-the-art data analysis strategy, artificial intelligence (AI) technologies have achieved remarkable success in medical imaging analysis. Numerous studies have shown great potential in automated quantification of lung abnormalities and severity prediction applying AI-based technologies (20–22).

Thus, the aim of this study is to assess CT features of COVID-19 patients with different smoking status using AI-based quantitative and visual scoring methods and to investigate changes of CT features in different disease states between the two groups.

## Materials and Methods

This retrospective study was approved by the Medical Ethical Committee (Approved Number.2020002), which waived the requirement for patients' informed consent.

### Patients

**Figure 1:**
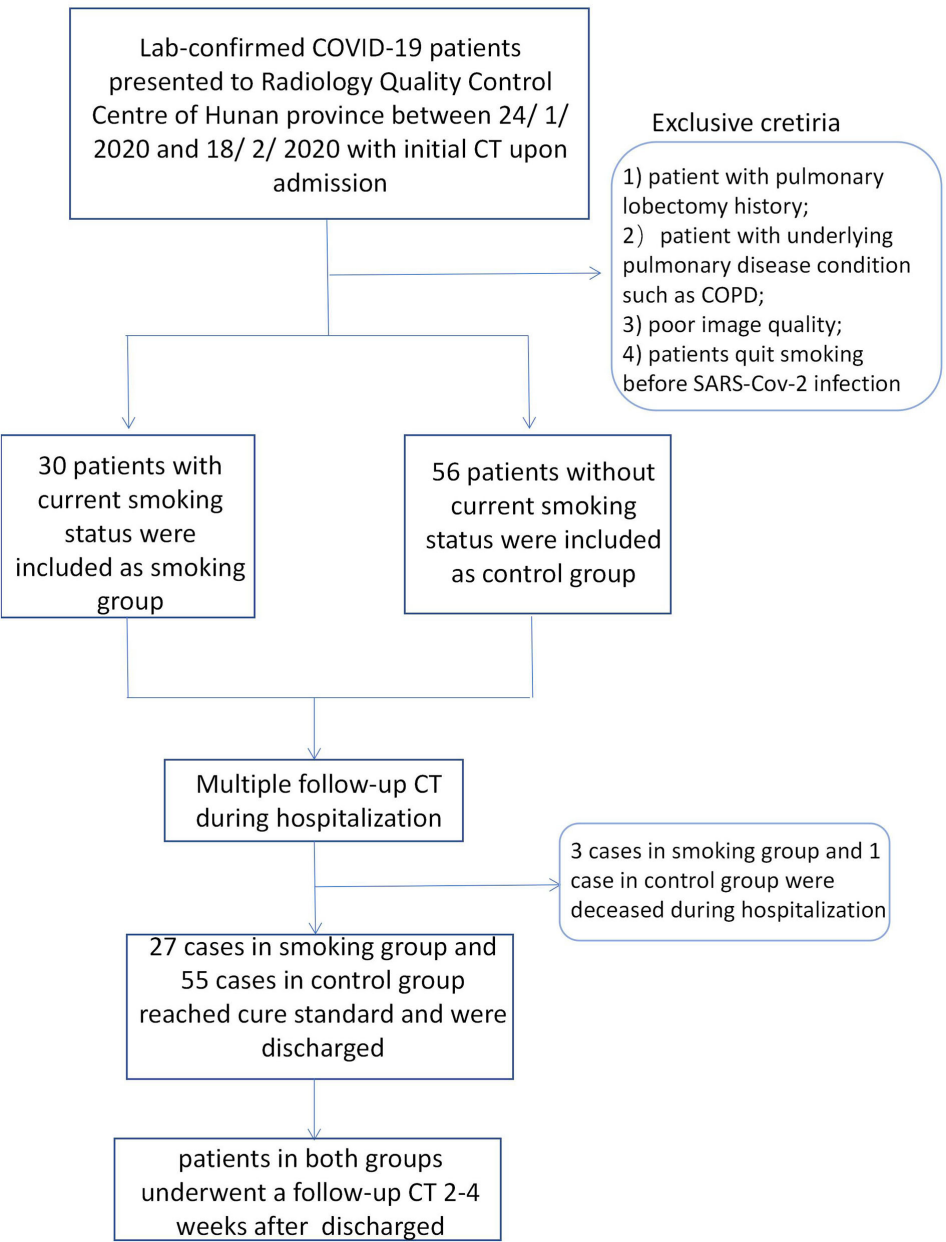
Patient flowchart.

We retrospectively searched the medical records of laboratory-confirmed COVID-19 patients with current smoking status from the Radiology Quality Control Center, Hunan, China, from January 24 to February 18, 2020. Patients who were current smokers or who quit smoking after SARS -Cov-2 infection were classed as having current smoking status. Current smoker was defined as someone who has smoked 100 cigarettes in his or her lifetime and who currently smokes cigarettes (23).The cigarette smoking intensity was quantified in pack-years (number of packs smoked per day multiplied by the number of years smoked) (24). Laboratory-confirmed non-smoking COVID-19 patients admitted during the same period were enrolled as a control group. Non-smokers were defined as patients who had never smoked, or who had smoked <100 cigarettes in his or her lifetime (23). Multiple CT images and clinical characteristics of all included patients were collected and analyzed. The diagnosis of COVID-19 was determined according to the following three methods: (1) isolation of COVID-19, (2) at least two positive results with real-time reverse-transcription polymerase chain reaction (RT-PCR) assay for COVID-19, or (3) a genetic sequence that matches COVID-19. The inclusion criteria of the smoking group was as follows: (1) patients with current smoking status or who quit smoking after infection, and (2) patients with multiple CT scans. The exclusion criteria were as follows: (1) patients with pulmonary lobectomy history; (2) patients with underlying pulmonary disease conditions such as COPD, (3) poor image quality, or (4) patients who quit smoking before SARS-Cov-2 infection. Finally, a total of 30 cases with current smoking history and 56 control cases were enrolled. Follow-up CT images during hospitalization for all patients were collected. The interval of follow-up CTs during hospitalization ranged from 2 to 7 days. All cases were treated strictly and followed the therapeutic principles based on the guidelines of COVID-19 (Trial Version 8) proposed by the China National Health Commission (25). The basic treatment included symptomatic treatment, recombinant human interferon α2b (aerosol inhalation), and antiviral treatment, such as lopinavir or ritonavir tablets (500 mg twice daily, orally) which were given to all confirmed cases. Corticosteroid treatment and antibiotic treatment were used where appropriate. Invasive mechanical ventilation treatment and extracorporeal membrane oxygenation (ECMO) was used for emergency cases (including severe and fetal cases). Patients who reached recovery standard according to COVID-19 guidelines (trial version 8) were discharged. Discharge criteria was as follows: (1) body temperature returned to normal for more than 3 days; (2) respiratory symptoms significantly relieved; (3) abnormal imaging findings substantially resolved; and (4) viral clearance, e.g., negative nucleic acid test for two consecutive respiratory pathogens (sampling interval ≥1 day). Recovered patients underwent another follow-up CT scan within 30 days after leaving hospital. The flowchart was shown in Figure 1.

### Imaging Analysis

Two thoracic radiologists (with 10 years of experience), who were blinded to smoking history and clinical data, reviewed initial and follow-up CT images independently and resolved discrepancies by consensus. All images were viewed on both lung (width, 1500 HU; level, −700 HU) and mediastinal (width, 350 HU; level, 40 HU) settings. The appearance of emphysema and change pattern of CT imaging were recorded. The CT image features at three time points were calculated and compared using both deep learning-based quantitative method and visual- based semi-quantitative method: initial CT upon admission, follow-up CT when discharged, and follow-up CT after being discharged.

#### Semi-Quantitative Assessment

A semi-quantitative assessment system was introduced to assess smoking-related interstitial lung changes (26, 27). Visual evaluation included a score of severity and a score of extent. The severity assessment was based on appreciation of five parenchymal abnormalities assumed to reflect increasing severity of lung involvement: ground-glass appearance (score 1), consolidation (score 2), septal thickening (score 3), reticulation (score 4), and honeycombing (score 5). The severity score thus ranged from 0 (no abnormality) to 15 (all abnormalities present). The extent score was obtained by counting the number of bronchopulmonary segments in which any of the previous abnormalities are observed: 1 to 3 segments involved implied a score of 1, 4 to 9 segments implied a score of 2, and more than 9 segments implied a score of 3. The extent score thus ranged from 0 (no abnormality in any segment) to 15 (all 5 abnormalities in more than 9 segments). Finally, severity and extent of disease scores were added to obtain a total score (range: 0–30).

#### Quantitative Assessment

The uAI software (uAI, Shanghai United Imaging Intelligence Co., Ltd.) was applied for quantitative CT feature assessment. This deep learning-based software could accurately segment the lung as well as the infection regions from chest CT images (28). This tool is based on deep learning, where a VB-net (22) is adopted to fulfill accurate segmentation of lung as well as infection regions from chest CT images. According to segmentation results, quantitative features, which are potentially related to COVID-19, are calculated. Specifically, the lung is segmented and divided into five lung lobes, i.e., superior/middle/inferior lobes of the right lung and superior/inferior lobes of the left lung, and 18 lung segments, with 10 segments in the right lung (denoted as RS1 – 10) and 8 segments in the left lung (denoted as LS1 – 8). In order to minimize the individual bias, we only included calibration features, including the ratio of infection volume to the whole lung, right/left lung, and each lobe/segment and within different HU ranges. Finally, 30 quantitative features were included in this study (Supplementary Table 1).

The change pattern of follow-up CT images were also investigated. We defined three imaging changes, namely no change, progress change, and improvement change, which we proposed in our previous study (16). No change referred to no obvious changes presented in chest CT. Progress change referred to the presence of new lesions or the presence of extent involvement area during the treatment. Improvement change referred to the continually absorbed abnormities.

## Statistical Analysis

Continuous variables with normal distribution were presented as mean ± standard deviation and compared by *Mann-Whitney U*-test. Continuous variables with non-normal distribution were presented as median (range) and compared by *Mann-Whitney U*-test. Categoric variables were presented as numbers (percentages) and were compared by *Fisher exact* test between smoking and non-smoking groups. The correlations between semi-quantitative results, quantitative results, and smoking intensity were analyzed using the Spearman analysis. Two-sided *p* < 0.05 was considered statistically significant. All statistical analyses were performed with SPSS software (version 19.0, IBM).

## Results

Among the 86 included patients, 30 (29 men, 1 women) were in the smoking group, and 56 (24 men, 32 women) were in the non-smoking group. The distribution of age and sex was shown in Table 1. There was no statistical difference regarding age between two groups. However, there were fewer females in the smoking group (*p* = 0.001). Clinical type of COVID-19 at baseline and the change patterns of follow-up CT images showed no statistical difference among the two groups (Table 1).

**Table 1:** Demographic and clinical features.

**Basic characteristics**	**Smoking group (*n* = 30)**	**Control group (*n* = 56)**	***P*-value**
Sex			**0.001**
Male	29 (97)	24 (43)	
Female	1 (3)	32 (57)	
Age (years)	50.83 ± 16.05	46.14 ± 13.34	0.152
Clinical type at baseline			0.075
Mild	2 (7)	4 (7)	
Common	22 (73)	49 (87)	
Severe	5 (17)	3 (5)	
Fatal	1 (3)	0	
Imaging features changes			0.056
Improvement change	12 (40)	37 (66)	
Progress change	3(10)	0	
Progressing and then improving change	11 (37)	15 (27)	
No change	4 (13)	4 (7)	
Presence of emphysema	12 (40)	3 (5)	**0.01**
Deceased cases	3 (10)	1 (1)	0.12

*The bold values stand for p < 0.05*.

### Evaluation of Initial CT

There were 14 cases (40%) in the smoking group where emphysema evidence was found on CT images, while only 3 cases (5%) showed emphysema in the control group (*p* = 0.001). Concerning semi-quantitative assessment for interstitial lung changes (Table 2), the scores of septal thickening [4.5 (0–5) vs. 0 (0–4)] and reticulation [0 (0–5.25) vs. 0 (0–0)] were significantly higher in the smoking group than in the control group (*p* < 0.05). The score of consolidation, however, was lower in the smoking group than in the control group [3 (0–3) vs. 3 (0–4), *p* < 0.05]. The total interstitial change scores were higher in the smoking group with statistical differences [7 (5–12.25) vs. 6 (5–7), *p* < 0.05]. Results with statistical significance of quantitative assessment of chest CT imaging upon admission were shown in Table 2. The infection ratio of whole lung and left lung were higher in the control group than in the smoking group (*p* < 0.05). To be more specific, the infection ratio in inferior lobe, LS6, LS7+8, LS9, and LS10 of left lung were higher in the control group with statistical differences. Infection ratio within HU (−750, −300) [0.75 (0.1–4.5) vs. 2.9 (1.0–6.1), *p* < 0.05] and within HU (−300, 49) [0.1 (0–0.95) vs. 0.85 (0.2–2.2), *p* < 0.05] were also higher in the control group than in the smoking group with statistical differences.

**Table 2:** CT features of initial CT.

**CT features**	**Smoking group (*n* = 30)**	**Control group (*n* = 56)**	***P*-value**
**Quantitative CT features**			
Infection ratio in the whole lung (%)	1.2 (0.1–5.5)	4.2 (1.50–7.35)	**0.031**
Infection ratio in inferior lobe of left lung (%)	0.3 (0–4.9)	6.1 (0.52–14.35)	**0.003**
Infection ratio in S6 of left lung (%)	0.05 (0–1.8)	1.8 (0–13.9)	**0.017**
Infection ratio in S7+8 of left lung (%)	0 (0–0.95)	0.45 (0–2.85)	**0.042**
Infection ratio in S9 of left lung (%)	0.05 (0–2.3)	6.6 (0.3–20.1)	**0.001**
Infection ratio in S10 of left lung (%)	0.05 (0–5.05)	3.5 (0.17–17.4)	**0.009**
Infection ratio within HU (−750, −300) (%)	0.75 (0.1–4.5)	2.9 (1.0–6.1)	**0.032**
Infection ratio within HU (−300, 49) (%)	0.1 (0–0.95)	0.85 (0.2–2.2)	**0.012**
**Interstitial changes score**			
GGO	3 (2–4)	3 (2–3)	0.711
Consolidation	3 (0–3)	3 (0–4)	**0.032**
Septal thickening	4.5 (0–5)	0 (0–4)	**0.001**
Reticulation	0 (0–5.25)	0 (0–0)	**0.001**
Honeycombing sign	0 (0–0)	0 (0–0)	0.173
Total score	7 (5–12.25)	6 (5–7)	**0.008**

*The bold values stand for p < 0.05*.

### Evaluation of Follow-Up CT When Discharged

There were three patients in the smoking group and one patient in the control group who unfortunately passed away during hospitalization. There were no statistical differences regarding deceased cases between two groups. The remaining 82 cases reached recovery standard and underwent follow-up CT scan when discharged. There were no statistical differences of hospitalization time between the smoking group and control group (19.37 ± 8.49 days vs. 18.47 ± 9.56 days, *p* = 0.68).

The total interstitial change scores when discharged showed no statistical insignificance between two groups (Table 3). The score of reticulation was significantly higher in the smoking group than in the control group [0.89 (0–0) vs. 0.09 (0–0), *p* = 0.02]. Results with statistical significance of quantitative CT features when discharged were shown in Table 3. The infection ratio of whole lung, the infection ratio of left lung, the infection ratio in inferior lobe, S6, S7+8, S9, and S10 of left lung, and infection ratio within HU (–,−750) as well as infection ratio within HU (−750, −300) were higher in the control group with statistical differences (*p* < 0.05).

**Table 3:** CT features of follow-up CT when discharged.

**CT features**	**Smoking group (*n* = 27)**	**Control group (*n* = 55)**	***P*-value**
Time from admission to discharged (days)	19.37 ± 8.49	18.47 ± 9.56	0.68
**Quantitative CT features**			
Infection ratio in the whole lung (%)	0 (0–1.7)	1.1 (0.2–3.8)	**0.016**
Infection ratio in the left lung (%)	0.1 (0–1.1)	0.6 (0.1–3.1)	**0.019**
Infection ratio in S6 of left lung (%)	0 (0–0.9)	0.7 (0–6.8)	**0.034**
Infection ratio in S7+8 of left lung (%)	0 (0–0.3)	0.2 (0–1.4)	**0.024**
Infection ratio in S9 of left lung (%)	0 (0–0.2)	0.7 (0–4.8)	**0.004**
Infection ratio in S10 of left lung (%)	0 (0–0.6)	0.6 (0.1–5.6)	**0.004**
Infection ratio within HU (–,−750) (%)	0 (0–0.2)	0.1 (0–0.4)	**0.029**
Infection ratio within HU (−750, −300) (%)	0 (0–1.2)	0.9 (0.1–2.8)	**0.010**
**Interstitial changes score**
GGO	3 (2, 3)	3 (2, 3)	0.364
Consolidation	0 (0–3)	0 (0–3)	0.398
Septal thickening	4 (0–5)	4 (0–4)	0.409
Reticulation	0.89 (0–0)	0.09 (0–0)	**0.02**
Honeycombing sign	0.2 (0–0)	0 (0–0)	0.154
Total score	7 (4–11)	7 (5–10)	0.85

*The bold values stand for p < 0.05*.

### Evaluation of Follow-Up CT 2-4 Weeks After Discharged

All recovered patients underwent CT scans 2–4 weeks after being discharged. The interval time of follow-up CT after being discharged is 28.8 ± 0.94 days for the smoking group and 27 ± 4.02 days for the control group. No statistical differences were discovered between two groups concerning follow-up time interval. Regarding the semi-quantitative features, only the score of septal thickening was shown to be higher in the smoking group than the control group (*p* = 0.007) 2–4 weeks after discharge (Table 4). There were plenty of quantitative CT features that were higher in the control group than in the smoking group at initial CT and when discharged. Interestingly, this trend turned adverse on follow-up CT 2–4 weeks after being discharged. The values of quantitative features were generally higher in the smoking group than in the control group, without statistical differences (Supplementary Table 1).

**Table 4:** CT features of follow-up CT after discharged 2–4 weeks.

**Interstitial changes score**	**Smoking group (*n* = 27)**	**Control group (*n* = 55)**	***P*-value**
Interval time of follow-up CT after discharged	28.8 ± 0.94	27 ± 4.02	0.097
GGO	2 (0–3)	0 (0–2)	0.443
Consolidation	0 (0–0)	0 (0–0)	0.528
Septal thickening	4 (0–4)	0 (0–4)	**0.007**
Reticulation	0 (0–0)	0 (0–0)	1
Honeycombing sign	0 (0–0)	0 (0–0)	1
Total score	4 (0–7)	0 (0–6)	0.069

*The bold value stand for p < 0.05*.

### Correlations Between Semi-Quantitative Results, Quantitative Results, and Cigarette Smoking Intensity

We investigated the relationships between interstitial changes score, quantitative CT features, and cigarette smoking intensity upon admission, when discharged, and 2–4 weeks after discharge. No significant correlations were found in terms of semi-quantitative or quantitative CT features (all *P* > 0.05) measured at all timepoints (Supplementary Table 4).

### Reproducibility

We reanalyzed CT features of both smoking group and control group cases upon admission for intra- and interobserver reproducibility of semi-quantitative assessment.

Reproducibility of semi-quantitative assessment was excellent within observers (intraclass correlation coefficient, 0.988; 95% confidence interval: 0.982, 0.992) and moderate between observers (intraclass correlation coefficient, 0.977; 95% confidence interval: 0.966, 0.984). No intra- or interobserver bias was noted.

## Discussion

**Figure 2:**
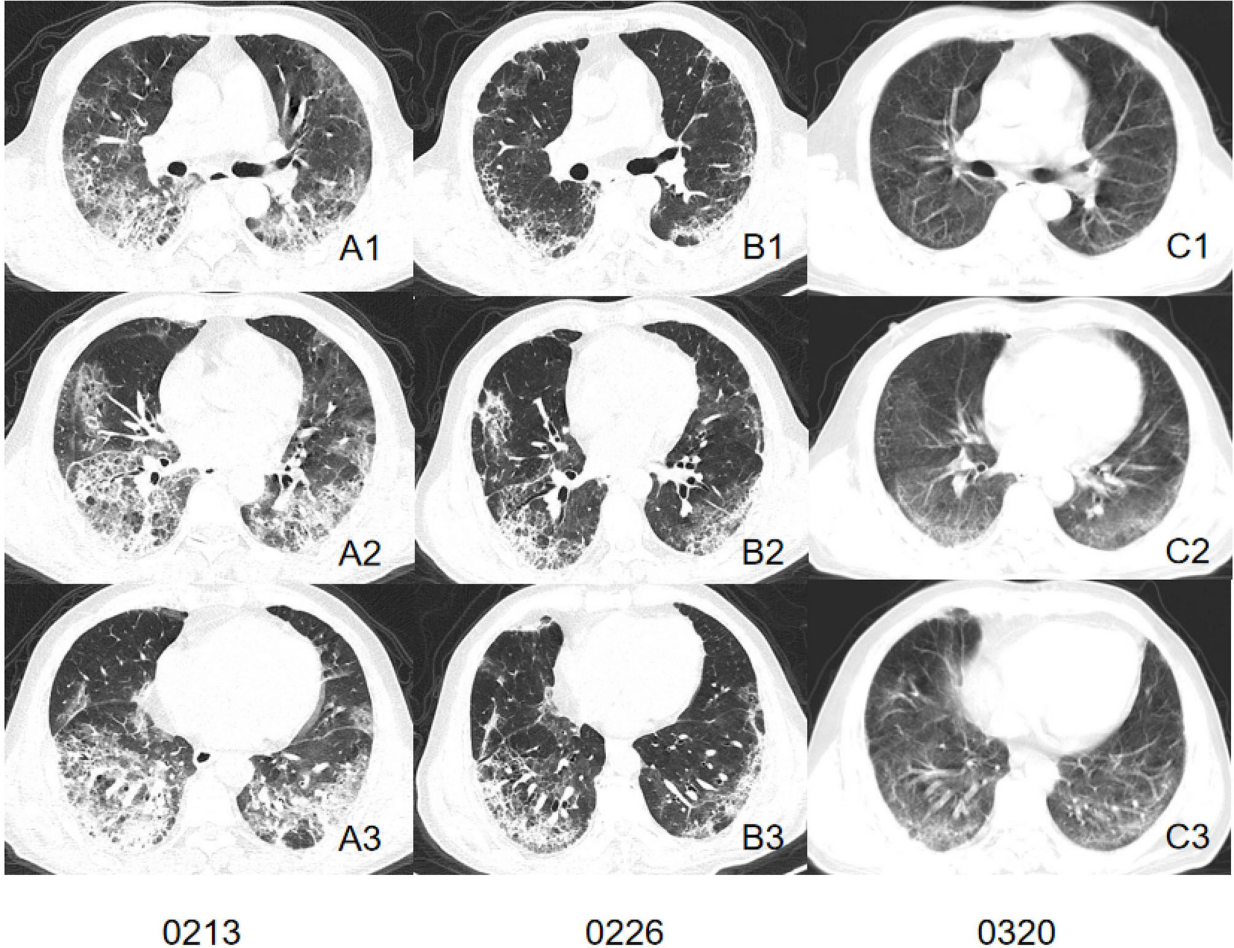
CT images of a patient in smoking group. Initial CT imaging **(A1–A3)** obtained on Feb 13 showed multiple GGO, mixed GGO, and consolidation with bilateral, peripheral, and predominately lower lung involvement. Interlobular septal thickening and reticulation were visible. Follow-up CT when discharged **(B1–B3)** showed absorbed lesion. Interstitial changes remained visible. A follow-up CT conducted 23 days after discharged **(C1–C3)** showed remain residual lesion with subpleural bands involved peripheral area of both lung.

**Figure 3:**
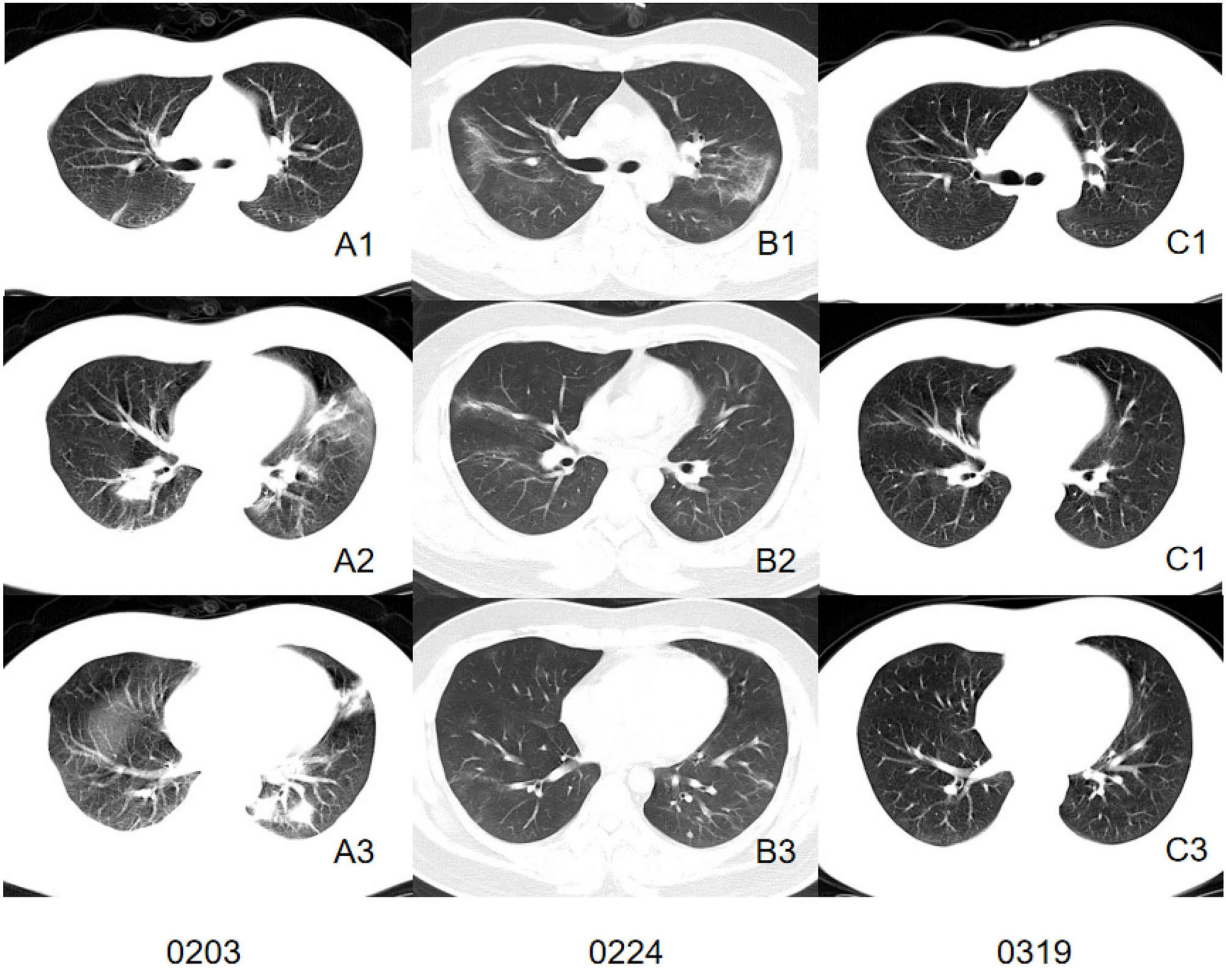
CT images of a patient in control group. Initial CT imaging **(A1–A3)** obtained on Feb 3 showed multiple mixed GGO and consolidation with bilateral, peripheral, and predominately lower lung involvement. Interlobular septal thickening and reticulation were not found. Follow-up CT when discharged **(B1–B3)** showed absorbed lesion. A follow-up CT conducted 24 days after discharged **(C1–C3)** showed completely absorbed lesion.

We comprehensively evaluated and analyzed the radiographic characteristics of 86 patients confirmed as having COVID-19 with or without current smoking status. The study demonstrated that patients with current smoking status presented more severe interstitial manifestations on CT images and may retain more residual lesion after being discharged (Figures 2, 3).

Typical CT features of COVID-19 were well-established by former studies (12, 30), which were consistent with our study. Both the smoking group and control group presented GGO or mixed GGO and consolidation with bilateral, peripheral, and predominately lower lung involvement. The post-mortem biopsy in COVID-19 patients reported pulmonary edema and hyaline membrane formation in both lungs, which might be the underlying pathological driver of GGO sign (31). However, several differences were found between patients confirmed as having COVID-19 pneumonia with or without current smoking status. Quantitative calculation of infection ratios of lung segments and infection ratios of regions within different HU ranges suggested that the control group presented a larger infection ratio than the smoking group and tended to present more GGO and consolidation involvement. However, visual assessment of interstitial changes showed that the smoking group presented more interstitial changes than the control group such as septal thickening and reticulation in the early stage of infection. These interesting phenomena suggest that patients without smoking history in our cohort presented larger lung involvement on chest CT images while the smoking group presented more severe interstitial changes, which might have contributed to more residual lesions. The exposure to smoke has been shown to modulate immune and adaptive immune responses when compared with those who had never smoked (32, 33). There was one female (38-year-old) in the control group who died during hospitalization due to a sudden virus-activated “cytokine-storm syndrome.” A previous study indicated that lack of exposure to smoke might partly contribute to a stronger immune response to SARS-Cov-2 infection and to the “cytokine-storm syndrome.” In this regard, we may assume that the immune system of a current smoker is more tolerant and less reactive compared to patients who have never smoked, which could explain the larger lung involvement presented in the control group. Interestingly, we discovered that GGOs of the smoking group tended to present an uneasily differentiated margin while the control group presented a more defined margin of GGOs. This might suggest the potential of further progress is expected. In contrast, a well-defined margin indicates that the lung manifestations were more restricted (34).

With cigarette exposure, smoking-related lung disease has already occurred before infection, including emphysema and fibrosis. In our study, initial CT scans consisted of former studies with more appearances of emphysema and interstitial changes. Furthermore, an animal study discovered that cigarette smoke disrupted lung endothelial barrier integrity and increased susceptibility to acute lung injury (35). From our results, we discovered that the initial response to SARS-Cov-2 infection for smoking group tended to present more as interstitial changes than the control group since the interstitial scores were higher for smoking patients. Studies have shown that the location and expression of ACE2 was dramatically affected by smoking status. A study conducted by Liu et al. discovered that smoking dramatically upregulates ACE2 expression in the secretory club cells of the bronchial epithelium, and exacerbates several pathological changes including oxidative stress, hypoxia, and inflammation (36). This might explain the three deceased cases in the smoking group who presented progressing CT change pattern and persistent hypoxemia.

As more recovered COVID-19 patients try to re-embrace their normal life, there is an urgent need to consider the long-term care needs of those affected by COVID-19. At the time of writing, the long-term effects on recovering patients remain unknown. Previous studies reported that the sequelae of patients infected with severe acute respiratory syndrome coronavirus (SARS) and middle eastern Respiratory syndrome coronavirus (MERS) infection were associated with persistent abnormal radiographic change, substantial impairment of exercise and functional capacity, and reduced quality of life (37). Our study discovered that patients without a smoking history tended to have a better response to treatment. This was proven by the assessment of imaging change patterns concerning follow-up CT during hospitalization, which indicated that the involvement of lesions was often shown to be continuously absorbing in the control group, while the majority of cases in the smoking group showed progressive lesion involvement or progressing before absorbing. Also, the follow-up CT after discharge indicated that patients with a smoking history were more likely to have persisting abnormal radiographic changes for a longer time. Therefore, for patients with a current smoking history, more attention should be paid during treatment to prevent disease progression. Also, more frequent follow-up and rehabilitation medical care should be focused on those patients to improve their life-quality after recovery from COVID-19.

Our study had several limitations. Firstly, the population of the smoking group was relatively small due to limited cases presented to our center. Therefore, the effects of smoking on COVID-19 patients might be quite variable in the cohort. The specific impact of different smoking status on COVID-19 disease is controversial. Hypotheses support both a potentially hazardous impact and a potentially protective effect (29). Our findings demonstrate that patients with a smoking history tend to have more interstitial lung change responses to SARS-Cov-2 infection and poor response to treatment during the course of the disease. Our conclusions need further investigation with a larger study population to be confirmed. We are unable to study differences between subgroups of different time periods of smoking history due to limited smoking cases. It might also explain that our analysis concerning correlations between semi-quantitative results, quantitative results, and smoking intensity showed no statistical difference. Since the number of patients with opposite outcomes is limited (with three deceased cases in the smoking group and one deceased case in the control group), the correlation analysis between CT features and different clinical outcomes is quite difficult to discuss in this cohort. Nevertheless, we are conducting a long-term follow-up study of recovered COVID-19 patients and we shall further investigate the correlations between quantifications of CT features and recovering progress. Secondly, the follow-up time after discharge was relatively short. A long-term follow-up is required. Lastly, more clinical information, such as lung function, should be included in future follow-up research.

In conclusion, patients with smoking history in our study tend to have more interstitial lung change responses to SARS-Cov-2 infection and poor response to treatment during the course of the disease. Follow-up CT images indicated that patients with a smoking history may retain more residual lesions. More support should be given for COVID-19 patients with smoking status during hospitalization and after being discharged.

## Data Availability Statement

The raw data supporting the conclusions of this article will be made available by the authors, without undue reservation.

## Ethics Statement

The studies involving human participants were reviewed and approved by Medical Ethical Committee of Second Xiangya Hospital (Approved Number. 2020002). Written informed consent for participation was not required for this study in accordance with the national legislation and the institutional requirements.

## Author Contributions

JL, XX, and SW designed the research and revised the paper. XX, WZ, and ZZ performed the research and acquired the data. All authors drafted and wrote the paper.

## Funding

This study has received funding by the Key Emergency Project of Pneumonia Epidemic of novel coronavirus infection (2020SK3006, 2020SK3014), Emergency Project of Prevention and Control for COVID-19 of Central South University (160260005), Foundation from Changsha Scientific and Technical bureau, China (kq2001001), National Natural Science Foundation of China (82102157), Hunan Provincial Natural Science Foundation of China (2021JJ40895), the Science and Technology Innovation Program of Hunan Province (2020SK53423), the Clinical Research Center For Medical Imaging In Hunan Province (2020SK4001), and The project of Changsha Science and Technology (kq1801115).

## Conflict of Interest

The authors declare that the research was conducted in the absence of any commercial or financial relationships that could be construed as a potential conflict of interest.

## Publisher's Note

All claims expressed in this article are solely those of the authors and do not necessarily represent those of their affiliated organizations, or those of the publisher, the editors and the reviewers. Any product that may be evaluated in this article, or claim that may be made by its manufacturer, is not guaranteed or endorsed by the publisher.
