# Supplementary material for: The Differences and Changes of Semi-Quantitative and Quantitative CT Features of Coronavirus Disease 2019 Pneumonia in Patients With or Without Smoking History
Source: Front Med (Lausanne). 2021 Sep 8;8:663514. doi: 10.3389/fmed.2021.663514 (PMC8455871; doi:10.3389/fmed.2021.663514)
Supplement: Supplementary file 1 [file Data_Sheet_1.doc]

Supplementary table1 30 Quantitative CT features of initial CT, follow-up CT when discharged and follow-up CT 2-4 weeks after discharged

|  | Initial CT | | | CT when discharged | | | CT after discharged 2-4 weeks | | |
| --- | --- | --- | --- | --- | --- | --- | --- | --- | --- |
| Quantitative CT features | Smoking group (n=30) | Control group (n=56) | P value | Smoking group (n=27) | Control group (n=55) | P value | Smoking group (n=27) | Control group (n=55) | P value |
| Infection ratio in the whole lung (%) | 1.2 (0.1-5.5) | 4.2 (1.5-7.35) | **0.031** | 0 (0-1.7) | 1.1 (0.2-3.8) | **0.016** | 0 (0-0.5) | 0 (0-0.2) | 0.978 |
| Infection ratio in the left lung (%) | 0.8 (0.1-4.2) | 3.6 (0.4-8.4) | 0.065 | 0.1 (0-1.1) | 0.6 (0.1-3.1) | **0.019** | 0 (0-0.1) | 0 (0-0.1) | 0.778 |
| Infection ratio in the right lung (%) | 1.2 (0-6.1) | 3.55 (0.32-11.1) | 0.073 | 0 (0-2.2) | 0.8 (0-3.1) | 0.083 | 0 (0-0.6) | 0 (0-0.1) | 0.478 |
| Infection ratio in superior  lobe of left lung (%) | 0.45 (0-2.8) | 0.2 (0-3.05) | 0.895 | 0 (0-0.6) | 0.1 (0-1) | 0.556 | 0 (0-0.2) | 0 (0-0.1) | 0.567 |
| Infection ratio in inferior  lobe of left lung (%) | 0.3 (0-4.9) | 6.1 (0.52-14.35) | **0.003** | 0 (0-1.3) | 0.9 (0.1-4) | **0.007** | 0 (0-0.1) | 0 (0-0.2) | 0.826 |
| Infection ratio in superior  lobe of right lung (%) | 0.1 (0-1.8) | 0.4 (0-3.4) | 0.339 | 0 (0-1.5) | 0.1 (0-2.1) | 0.487 | 0 (0-0.1) | 0 (0-0) | 0.436 |
| Infection ratio in middle  lobe of right lung (%) | 0.1 (0-2.0) | 0.2 (0-2.6) | 0.473 | 0 (0-0.2) | 0 (0-0.5) | 0.413 | 0 (0-0.1) | 0 (0-0) | 0.521 |
| Infection ratio in inferior  lobe of right  lung (%) | 0.65 (0-14.4) | 4.9 (0.6-20.2) | 0.072 | 0 (0-2.2) | 1.2 (0-6.6) | 0.140 | 0 (0-0.7) | 0 (0-0.1) | 0.437 |
| Infection ratio in S1+2 of left lung (%) | 0.2 (0-4.6) | 0.1 (0-2.5) | 0.404 | 0 (0-0.2) | 0 (0-1.1) | 0.300 | 0 (0-0.1) | 0 (0-0.1) | 0.944 |
| Infection ratio in S3 of left lung (%) | 0 (0-1.8) | 0 (0-1.15) | 0.642 | 0 (0-0.2) | 0 (0-0.3) | 0.453 | 0 (0-0) | 0 (0-0.1) | 0.621 |
| Infection ratio in S4 of left lung (%) | 0 (0-2.9) | 0.05 (0-3.4) | 0.525 | 0 (0-0.3) | 0 (0-0.8) | 0.476 | 0 (0-0) | 0 (0-0) | 0.503 |
| Infection ratio in S5 of left lung (%) | 0.1 (0-4.0) | 0.1 (0-2.4) | 0.581 | 0 (0-0.2) | 0 (0-0.3) | 0.826 | 0 (0-0.2) | 0 (0-0.1) | 0.235 |
| Infection ratio in S6 of left lung (%) | 0.05 (0-1.8) | 1.8 (0-13.9) | **0.017** | 0 (0-0.9) | 0.7 (0-6.8) | **0.034** | 0 (0-0.1) | 0 (0-0.1) | 0.381 |
| Infection ratio in S7+8 of left lung (%) | 0 (0-0.95) | 0.45 (0-2.85) | **0.042** | 0 (0-0.3) | 0.2 (0-1.4) | **0.024** | 0 (0-0) | 0 (0-0.1) | 0.392 |
| Infection ratio in S9 of left lung (%) | 0.05 (0-2.3) | 6.6 (0.3-20.1) | **0.001** | 0 (0-0.2) | 0.7 (0-4.8) | 0.004* | 0 (0-0) | 0 (0-0.2) | 0.197 |
| Infection ratio in S10 of left lung (%) | 0.05 (0-5.05) | 3.5 (0.17-17.4) | **0.009** | 0 (0-0.6) | 0.6 (0.1-5.6) | 0.004* | 0 (0-0) | 0 (0-0.2) | 0.110 |
| Infection ratio in S1 of right lung (%) | 0 (0-0.7) | 0 (0-1.5) | 0.736 | 0(0-0.2) | 0 (0-0.6) | 0.315 | 0 (0-0) | 0 (0-0) | 0.505 |
| Infection ratio in S2 of right lung (%) | 0.05 (0-2.6) | 0.2 (0-7.9) | 0.484 | 0(0-1.7) | 0 (0-1.8) | 0.757 | 0 (0-0.1) | 0 (0-0) | 0.138 |
| Infection ratio in S3 of right lung (%) | 0 (0-0.5) | 0.05 (0-2.02) | 0.426 | 0(0-0.3) | 0 (0-0.8) | 0.217 | 0 (0-0) | 0 (0-0) | 0.856 |
| Infection ratio in S4 of right lung (%) | 0 (0-1.8) | 0.1 (0-2.9) | 0.502 | 0(0-0.2) | 0 (0-0.7) | 0.515 | 0 (0-0.1) | 0 (0-0) | 0.393 |
| Infection ratio in S5 of right lung (%) | 0.05 (0-2.1) | 0.1 (0-1.30) | 0.720 | 0(0-0.1) | 0 (0-0.1) | 0.916 | 0 (0-0) | 0 (0-0) | 0.679 |
| Infection ratio in S6 of right lung (%) | 0.1 (0-7.4) | 4.9 (0-23.1) | 0.125 | 0(0-3.7) | 0.4 (0-6.4) | 0.183 | 0 (0-0.2) | 0 (0-0.1) | 0.607 |
| Infection ratio in S7 of right lung (%) | 0 (0-1.0) | 0.2 (0-3.7) | 0.171 | 0(0-0.2) | 0 (0-0.1) | 0.872 | 0 (0-0) | 0 (0-0) | 0.429 |
| Infection ratio in S8 of right lung (%) | 0.2 (0-4.0) | 1.5 (0-7.7) | 0.427 | 0 (0-0.5) | 0.2 (0-1.2) | 0.265 | 0 (0-0.1) | 0 (0-0) | 0.500 |
| Infection ratio in S9 of right lung (%) | 0.7 (0-29.0) | 7.6 (0-34.4) | 0.341 | 0 (0-3.6) | 0.2 (0-6.2) | 0.684 | 0 (0-1.7) | 0 (0-0) | 0.194 |
| Infection ratio in S10 of right lung (%) | 0.05 (0-20.9) | 6.4 (0.2-22.6) | 0.065 | 0 (0-2.9) | 0.3 (0-5.2) | 0.167 | 0 (0-0.2) | 0 (0-0.2) | 0.434 |
| Infection ratio within HU (-, -750) (%) | 0 (0-0.2) | 0.05 (0-0.1) | 0.842 | 0 (0-0.2) | 0.1 (0-0.4) | 0.029* | 0 (0-0) | 0 (0-0) | 0.926 |
| Infection ratio within HU [-750, -300) (%) | 0.75 (0.1-4.5) | 2.9 (1.0-6.1) | **0.032** | 0 (0-1.2) | 0.9 (0.1-2.8) | 0.010* | 0 (0-0.3) | 0 (0-0.1) | 0.870 |
| Infection ratio within HU [-300,49) (%) | 0.1 (0-0.95) | 0.85 (0.2-2.2) | **0.012** | 0 (0-0.3) | 0.1 (0-0.3) | 0.171 | 0 (0-0) | 0 (0-0) | 0.555 |
| Infection ratio within HU [50+) (%) | 0 (0-0) | 0 (0-0) | 0.669 | 0 (0-0) | 0 (0-0) | 0.151 | 0 (0-0) | 0 (0-0) | 0.484 |

Supplementary Table2 CT features of initial CT

| CT features | Smoking group (n=30) | Control group (n=56) | P value |
| --- | --- | --- | --- |
| Quantitative CT features |  |  |  |
| Infection ratio in the whole lung (%) | 0.8 (0.1-4.8) | 4.2 (1.80-7.40) | **0.03** |
| Infection ratio in the left lung (%) | 0.8 (0.1-2.1) | 3.7 (0.4-8.5) | **0.01** |
| Infection ratio in the right lung (%) | 0.6 (0-3.9) | 3.6 (0.4-11.4) | **0.01** |
| Infection ratio in inferior  lobe of left lung (%) | 0.2 (0-1.9) | 6.2 (0.5-14.6) | **0.001** |
| Infection ratio in S6 of left lung (%) | 0 (0-1.7) | 2.2 (0-14.9) | **0.012** |
| Infection ratio in S7+8 of left lung (%) | 0 (0-0.2) | 0.5 (0-3.1) | **0.004** |
| Infection ratio in S9 of left lung (%) | 0(0-0.9) | 6.6 (0.3-20.5) | **0.001** |
| Infection ratio in S10 of left lung (%) | 0 (0-3.9) | 4.0 (0.4-18.0) | **0.001** |
| Infection ratio in S6 of right lung (%) | 0 (0-1.6) | 5.5 (0-23.1) | **0.03** |
| Infection ratio in S7 of right lung (%) | 0 (0-0.4) | 0.2 (0-3.8) | **0.03** |
| Infection ratio in S10 of right lung (%) | 0 (0-12.6) | 7.0 (0.2-23.0) | **0.006** |
| Infection ratio within HU [-750,-300) (%) | 0.6 (0-2.8) | 2.9 (1.1-6.3) | **0.002** |
| Infection ratio within HU [-300,49) (%) | 0.1 (0-0.5) | 0.9 (0.3-2.2) | **0.003** |
| Interstitial changes score |  |  |  |
| GGO | 3 (2-3) | 3 (2-3) | 0.975 |
| Consolidation | 3 (0-3) | 3 (0-4) | **0.016** |
| Septal thickening | 0 (0-5) | 0 (0-4) | **0.008** |
| Reticulation | 0 (0-5.25) | 0 (0-0) | **0.001** |
| Honeycombing sign | 0 (0-0) | 0 (0-0) | 1 |
| Total score | 6 (5-12) | 6 (5-7) | 0.521 |

Supplementary Table3 CT features of follow-up CT when discharged

| CT features | Smoking group (n=27) | Control group (n=55) | P value |
| --- | --- | --- | --- |
| Quantitative CT features |  |  |  |
| Infection ratio in the whole lung (%) | 0 (0-1.7) | 1.1 (0.2-3.8) | **0.016** |
| Infection ratio in the left lung (%) | 0.1 (0-1.1) | 0.6 (0.1-3.1) | **0.019** |
| Infection ratio in S6 of left lung (%) | 0 (0-0.9) | 0.7 (0-6.8) | **0.034** |
| Infection ratio in S7+8 of left lung (%) | 0 (0-0.3) | 0.2 (0-1.4) | **0.024** |
| Infection ratio in S9 of left lung(%) | 0 (0-0.2) | 0.7 (0-4.8) | **0.004** |
| Infection ratio in S10 of left lung (%) | 0 (0-0.6) | 0.6 (0.1-5.6) | **0.004** |
| Infection ratio within HU (-,-750) (%) | 0 (0-0.2) | 0.1 (0-0.4) | **0.029** |
| Infection ratio within HU [-750, -300) (%) | 0 (0-1.2) | 0.9 (0.1-2.8) | **0.010** |
| Interstitial changes score |  |  |  |
| GGO | 3 (2-3) | 3 (2-3) | 0.364 |
| Consolidation | 0 (0-3) | 0 (0-3) | 0.398 |
| Septal thickening | 4 (0-5) | 4 (0-4) | 0.409 |
| Reticulation | 0.89 (0-0) | 0.09 (0-0) | **0.02** |
| Honeycombing sign | 0.2 (0-0) | 0 (0-0) | 0.154 |
| Total score | 7 (4-11) | 7 (5-10) | 0.85 |

Supplementary Table4 Correlations between semi-quantitative results, quantitative results and cigarette smoking intensity

|  | Initial CT | | CT when discharged | | |  | | CT after discharged 2-4 weeks | | |
| --- | --- | --- | --- | --- | --- | --- | --- | --- | --- | --- |
| CT features | r | P value | | r | P value | | r | | P value |  |
| Quantitative features |  |  | |  |  | |  | |  |  |
| Infection ratio in the whole lung (%) | 0.081 | 0.699 | | -0.025 | 0.907 | | -0.039 | | 0.854 |  |
| Infection ratio in the left lung (%) | -0.057 | 0.785 | | -0.029 | 0.890 | | -0.038 | | 0.857 |  |
| Infection ratio in the right lung (%) | 0.080 | 0.705 | | 0.102 | 0.627 | | 0.121 | | 0.565 |  |
| Infection ratio in superior  lobe of left lung (%) | -0.104 | 0.621 | | 0.060 | 0.775 | | -0.030 | | 0.887 |  |
| Infection ratio in inferior  lobe of left lung (%) | -0.005 | 0.982 | | -0.059 | 0.778 | | -0.029 | | 0.892 |  |
| Infection ratio in superior  lobe of right lung (%) | -0.073 | 0.729 | | -0.052 | 0.805 | | 0.126 | | 0.548 |  |
| Infection ratio in middle  lobe of right lung (%) | -0.030 | 0.885 | | 0.066 | 0.754 | | 0.214 | | 0.305 |  |
| Infection ratio in inferior  lobe of right  lung (%) | 0.017 | 0.935 | | 0.083 | 0.693 | | 0.129 | | 0.540 |  |
| Infection ratio in S1+2 of left lung (%) | -0.078 | 0.712 | | 0.210 | 0.313 | | 0.099 | | 0.638 |  |
| Infection ratio in S3 of left lung (%) | 0.162 | 0.439 | | 0.026 | 0.902 | | 0.008 | | 0.969 |  |
| Infection ratio in S4 of left lung (%) | -0.114 | 0.587 | | 0.036 | 0.866 | | 0.128 | | 0.541 |  |
| Infection ratio in S5 of left lung (%) | -0.236 | 0.255 | | 0.048 | 0.819 | | 0.095 | | 0.653 |  |
| Infection ratio in S6 of left lung (%) | 0.045 | 0.833 | | 0.005 | 0.981 | | 0.015 | | 0.943 |  |
| Infection ratio in S7+8 of left lung (%) | -0.076 | 0.719 | | -0.019 | 0.927 | | -0.231 | | 0.267 |  |
| Infection ratio in S9 of left lung (%) | 0.022 | 0.917 | | 0.345 | 0.091 | | 0.208 | | 0.317 |  |
| Infection ratio in S10 of left lung (%) | 0.014 | 0.945 | | 0.222 | 0.286 | | 0.094 | | 0.654 |  |
| Infection ratio in S1 of right lung (%) | -0.181 | 0.386 | | 0.162 | 0.440 | | 0.005 | | 0.980 |  |
| Infection ratio in S2 of right lung (%) | 0.182 | 0.384 | | 0.120 | 0.569 | | 0.224 | | 0.283 |  |
| Infection ratio in S3 of right lung (%) | -0.314 | 0.127 | | -0.224 | 0.281 | | 0.014 | | 0.946 |  |
| Infection ratio in S4 of right lung (%) | 0.086 | 0.682 | | 0.176 | 0.401 | | 0.135 | | 0.519 |  |
| Infection ratio in S5 of right lung (%) | -0.146 | 0.486 | | 0.039 | 0.853 | | -0.003 | | 0.990 |  |
| Infection ratio in S6 of right lung (%) | 0.183 | 0.381 | | 0.137 | 0.514 | | 0.180 | | 0.388 |  |
| Infection ratio in S7 of right lung (%) | 0.089 | 0.673 | | 0.229 | 0.271 | | 0.105 | | 0.618 |  |
| Infection ratio in S8 of right lung (%) | 0.067 | 0.749 | | 0.275 | 0.183 | | 0.220 | | 0.292 |  |
| Infection ratio in S9 of right lung (%) | -0.005 | 0.982 | | 0.054 | 0.798 | | 0.089 | | 0.671 |  |
| Infection ratio in S10 of right lung (%) | -0.010 | 0.961 | | 0.187 | 0.371 | | 0.102 | | 0.628 |  |
| Infection ratio within HU (-, -750) (%) | 0.321 | 0.117 | | 0.206 | 0.324 | | 0.324 | | 0.114 |  |
| Infection ratio within HU [-750, -300) (%) | 0.106 | 0.615 | | 0.021 | 0.921 | | 0.060 | | 0.777 |  |
| Infection ratio within HU [-300,49) (%) | 0.036 | 0.864 | | 0.199 | 0.341 | | -0.053 | | 0.800 |  |
| Infection ratio within HU [50+) (%) | 0.072 | 0.734 | | 0.129 | 0.538 | | 0 | | 0 |  |
| Interstitial changes score |  |  | |  |  | |  | |  |  |
| GGO | -0.27 | 0.899 | | -0.29 | 0.889 | | 0.134 | | 0.523 |  |
| Consolidation | -0.9 | 0.87 | | 0.15 | 0.475 | | 0 | | 0 |  |
| Septal thickening | 0.164 | 0.435 | | 0.187 | 0.37 | | 0.134 | | 0.524 |  |
| Reticulation | 0.144 | 0.494 | | 0.237 | 0.254 | | 0 | | 0 |  |
| Honeycombing sign | 0 | 0 | | -0.129 | 0.54 | | 0 | | 0 |  |
| Total score | 0.042 | 0.842 | | 0.089 | 0.671 | | 0.109 | | 0.604 |  |
